# Taxonomic interpretation of chromosomal and mitochondrial DNA variability in the species complex close to Polyommatus (Agrodiaetus) dama (Lepidoptera, Lycaenidae)

**DOI:** 10.3897/zookeys.538.6559

**Published:** 2015-11-19

**Authors:** Nazar A. Shapoval, Vladimir A. Lukhtanov

**Affiliations:** 1 Department of Karyosystematics, Zoological Institute of Russian Academy of Sciences, Universitetskaya nab. 1, St. Petersburg 199034, Russia; 2 Department of Entomology, Faculty of Biology, St. Petersburg State University, Universitetskaya nab. 7/9, St. Petersburg 199034, Russia

**Keywords:** *COI*, Iran, karyotype, molecular marker, chromosome number

## Abstract

In this paper, by using combination of molecular and chromosomal markers, populations of Polyommatus (Agrodiaetus) karindus (Riley, 1921) from north-west and central Iran are analyzed. It has been found that taxon usually identified as Polyommatus (Agrodiaetus) karindus is represented in Iran by two geographically separated groups of individuals, strongly differentiated by their karyotypes and mitochondrial haplotypes. It is demonstrated that populations from NW Iran have the haploid chromosome number n = 68, while the haploid chromosome number of Polyommatus (Agrodiaetus) karindus from central Iran is found to be n = 73. Phylogenetic analysis revealed that these groups also differ by at least eight nucleotide substitutions in a 690 bp fragment of the mitochondrial *COI* gene and form separated groups of clusters in Bayesian inference tree. Thus, population entities from central Iran are described here as a new subspecies Polyommatus (Agrodiaetus) karindus saravandi **ssp. n.** Strong chromosomal and molecular differentiation are confirmed between Polyommatus (Agrodiaetus) karindus and its sister species, Polyommatus (Agrodiaetus) dama (Staudinger, 1892).

## Introduction

*Agrodiaetus* Hübner, 1822 is the most species-rich subgenus within the genus *Polyommatus* Latreille, 1804 (Talavera et al. 2013a, Lukhtanov et al. 2015a). It consists of approximately 130 species distributed in the western Palearctic (Vila et al. 2010, Lukhtanov et al. 2008, 2014, Vershinina and Lukhtanov 2010, Przybyłowicz et al. 2014, Lukhtanov and Tikhonov 2015). Today *Agrodiaetus* has become a model group in studies of speciation (Lukhtanov et al. 2005, 2015b), intraspecific differentiation (Dincă et al. 2013, Przybyłowicz et al. 2014, Lukhtanov et al. 2015a), and rapid karyotype evolution (Lukhtanov et al., 2005, Kandul et al. 2007). From the point of view of taxonomy, *Agrodiaetus* is a very complicated group. Many *Agrodiaetus* taxa display extremely similar phenotype (Hesselbarth et al. 1995) and, in contrast to other Lepidoptera taxa, genitalia offer only few distinctive features. Furthermore, many taxa represent allopatric populations which differ only slightly in morphology, and a conclusion on their status as distinct species or subspecies is controversial and can be misleading (Wiemers 2003, Lukhtanov et al. 2015a). This resulted in description of numerous polytypic species based on geographic distribution and classic morphological characters (Forster 1956, 1960a, b, 1961).

In particular, Polyommatus (Agrodiaetus) dama (Staudinger, 1892) was traditionally regarded as a polytypic species that included two subspecies: Polyommatus (Agrodiaetus) dama dama (Staudinger, 1892) (orig. comb. *Lycaena Dama*) and Polyommatus (Agrodiaetus) dama karindus (Riley, 1921) (orig. comb. Lycaena dama subsp. karinda). Polyommatus (Agrodiaetus) dama dama has only been found in South Anatolia (a few localities in Malatya, Maraş, and Mardin provinces (Turkey), while Polyommatus (Agrodiaetus) dama karindus distribution range is restricted to Zagros Mountains in Iran.

The karyotype studies of de Lesse (1957, 1959a, b, c, d, 1960a, b, 1961, 1962a, b, 1963a, b, 1964, 1966, 1968) revealed that *Agrodiaetus* species exhibit a wide diversity of karyotypes. Karyotyping may provide necessary diagnostic character for many *Agrodiaetus* species, and therefore become an important requirement for describing new taxa (de Lesse 1960a, b, Lukhtanov and Dantchenko 2002, 2003, Lukhtanov et al. 2008). Karyological investigations showed strong chromosomal differentiation between Turkish and Iranian populations of Polyommatus (Agrodiaetus) dama s. l.. De Lesse (1959a) described karyotype of Polyommatus (Agrodiaetus) dama dama from Kahramanmaraş and Olivier et al. (1999) confirmed his results from the type locality Malatya. It has an asymmetric karyotype with n = 41 chromosomes, about eleven of them are large, gradually decreasing in size, the others medium–sized; whereas the karyotype of Iranian taxon was determined as n = 68 (Wiemers 2003). Thus, on the basis of karyotype studies, Polyommatus (Agrodiaetus) dama s. l. was split into two species, Polyommatus (Agrodiaetus) dama and Polyommatus (Agrodiaetus) karindus, that can be characterized by species-specific haploid chromosome numbers.

However, the chromosome number of Polyommatus (Agrodiaetus) karindus was determined only for one population from NW Iran (Saqqez, Kordestan Province) (Wiemers 2003). Further investigations showed that Iranian species Polyommatus (Agrodiaetus) karindus has complicated genetic and phylogeographic structure (Lukhtanov et al. 2015b). Here a combination of molecular mitochondrial (*COI*) and nuclear chromosomal (karyotype) markers are used to analyze different Iranian populations of Polyommatus (Agrodiaetus) karindus. Our study demonstrates that butterflies from central Iran strongly differentiated by their karyotypes and mitochondrial haplotypes from NW Iranian populations. Thus, population entities from central Iran are described here as a separate subspecies Polyommatus (Agrodiaetus) karindus saravandi ssp. n.

## Material and methods

### Specimens sampling

**Figure 1.**
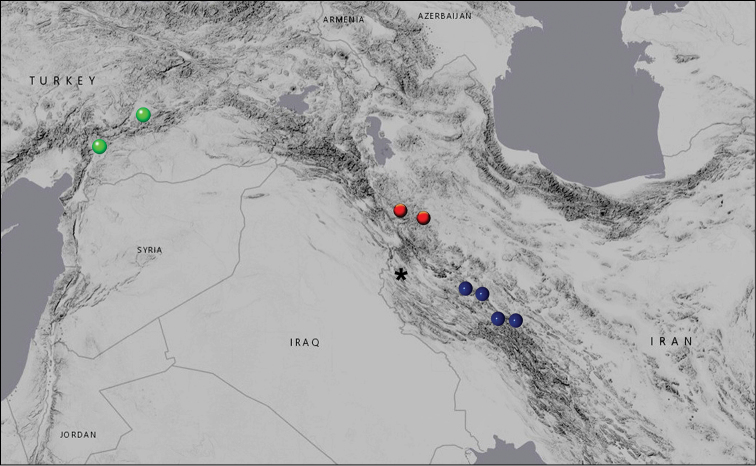
Distribution ranges of Polyommatus (Agrodiaetus) dama (green circles), Polyommatus (Agrodiaetus) karindus karindus (red circles) and Polyommatus (Agrodiaetus) karindus saravandi (blue circles). The asterisk indicates the type locality of Polyommatus (Agrodiaetus) karindus karindus.

The butterflies were collected in the period of 2007–2014 in Iran (list of collected specimens is given in Table 1). In north–west Iran we collected material in two localities: 1) in the mountain range between Saqqez and Baneh (30–40 km SW of Saqqez), and 2) in the vicinity of Dare Dozdan (30–40 km W of Divandarreh). In central Iran we collected butterflies in the vicinity of Vennai (18 km W of Borujerd), in the vicinity of Saravand (15 km SE of Dorud), in the vicinity of Nahavand and in the vicinity of Darreh Takht (35 km NE of Dorud) (information about sampling localities is given in Figure 1 and Table 1).

**Table 1.** List of studied material (129 specimens) with information on karyotype (48 specimens) and *COI* sequences (54 specimens). Collectors: V. Lukhtanov (VL), N. Shapoval (NS) and A. Barabanov (AB).

Species	Sex	Sample ID	Chromosome number (n)	COI	GenBank number	Province	Locality and coordinates	Altitude	Date	Collectors
*karindus*	M	E391	70			Kordestan	ca. 40 km SW Saqqez 36°06.18'N; 046°00.27'E	1725 m	29 July 2004	VL
*karindus*	M	E399	68			Kordestan	ca. 40 km SW Saqqez 36°06.18'N; 046°00.27'E	1725 m	29 July 2004	VL
*karindus*	M	E400	68			Kordestan	ca. 40 km SW Saqqez 36°06.18'N; 046°00.27'E	1725 m	29 July 2004	VL
*karindus*	M	E402	68			Kordestan	ca. 40 km SW Saqqez 36°06.18'N; 046°00.27'E	1725 m	29 July 2004	VL
*karindus*	M	Z726	68	h02/GH5	KT582701	Kordestan	ca. 40 km SW Saqqez 36°05.97'N; 045°59.63'E	1720m	30 July 2007	VL & NS
*karindus*	M	Z727	68	h03/GH5	KT582702	Kordestan	ca. 40 km SW Saqqez 36°05.97'N; 045°59.63'E	1720m	30 July 2007	VL & NS
*karindus*	M	Z729	68			Kordestan	ca. 40 km SW Saqqez 36°05.97'N; 045°59.63'E	1720m	30 July 2007	VL & NS
*karindus*	M	Z749		h01/GH5	KT582703	Kordestan	ca. 40 km SW Saqqez 36°04.82'N; 045°58.88'E	1880m	31 July 2007	VL & NS
*karindus*	M	Z750		h01/GH5	KT582704	Kordestan	ca. 40 km SW Saqqez 36°04.82'N; 045°58.88'E	1880m	31 July 2007	VL & NS
*karindus*	M	Z753	68	h01/GH5	KT582705	Kordestan	ca. 40 km SW Saqqez 36°04.82'N; 045°58.88'E	1880m	31 July 2007	VL & NS
*karindus*	M	Z800	68	h01/GH5	KT582706	Kordestan	ca. 40 km SW Saqqez 36°04.09'N; 045°58.82'E	2050m	31 July 2007	VL & NS
*karindus*	M	Z809	68	h01/GH5	KT582707	Kordestan	ca. 40 km SW Saqqez 36°04.09'N; 045°58.82'E	2050m	31 July 2007	VL & NS
*karindus*	M	Z820	68	h01/GH5	KT582708	Kordestan	ca. 40 km SW Saqqez 36°04.09'N; 045°58.82'E	2050m	31 July 2007	VL & NS
*karindus*	M	Z843	68	h01/GH5	KT582709	Kordestan	ca. 40 km SW Saqqez 36°04.64'N; 045°59.16'E	1920–1950m	1 August 2007	VL & NS
*karindus*	M	Z845	69	h01/GH5	KT582710	Kordestan	ca. 40 km SW Saqqez 36°04.64'N; 045°59.16'E	1920–1950m	1 August 2007	VL & NS
*karindus*	M	W253	68			Kordestan	ca. 40 km SW Saqqez 36°03.00'N; 045°58.54'E	2027m	29 July 2009	VL & NS
*karindus*	M	W254	68			Kordestan	ca. 40 km SW Saqqez 36°03.00'N; 045°58.54'E	2027m	29 July 2009	VL & NS
*karindus*	M	W259	68			Kordestan	ca. 40 km SW Saqqez 36°03.00'N; 045°58.54'E	2027m	29 July 2009	VL & NS
*karindus*	M	W271	68			Kordestan	ca. 40 km SW Saqqez 36°04.39'N; 045°59.06'E	1869m	29 July 2009	VL & NS
*karindus*	M	W272	68			Kordestan	ca. 40 km SW Saqqez 36°04.39'N; 045°59.06'E	1869m	29 July 2009	VL & NS
*karindus*	M	W273	68			Kordestan	ca. 40 km SW Saqqez 36°04.39'N; 045°59.06'E	1869m	29 July 2009	VL & NS
*karindus*	M	W274	68			Kordestan	ca. 40 km SW Saqqez 36°04.39'N; 045°59.06'E	1869m	29 July 2009	VL & NS
*karindus*	M	W275	68			Kordestan	ca. 40 km SW Saqqez 36°04.39'N; 045°59.06'E	1869m	29 July 2009	VL & NS
*karindus*	M	W276	68			Kordestan	ca. 40 km SW Saqqez 36°04.39'N; 045°59.06'E	1869m	29 July 2009	VL & NS
*karindus*	M	W277	68			Kordestan	ca. 40 km SW Saqqez 36°04.39'N; 045°59.06'E	1869m	29 July 2009	VL & NS
*karindus*	M	W278	68			Kordestan	ca. 40 km SW Saqqez 36°04.39'N; 045°59.06'E	1869m	29 July 2009	VL & NS
*karindus*	M	W279	68			Kordestan	ca. 40 km SW Saqqez 36°04.39'N; 045°59.06'E	1869m	29 July 2009	VL & NS
*karindus*	M	W280	68			Kordestan	ca. 40 km SW Saqqez 36°04.39'N; 045°59.06'E	1869m	29 July 2009	VL & NS
*karindus*	M	W281	68			Kordestan	ca. 40 km SW Saqqez 36°04.39'N; 045°59.06'E	1869m	29 July 2009	VL & NS
*karindus*	M	W282	68			Kordestan	ca. 40 km SW Saqqez 36°04.39'N; 045°59.06'E	1869m	29 July 2009	VL & NS
*karindus*	M	W283	68			Kordestan	ca. 40 km SW Saqqez 36°04.39'N; 045°59.06'E	1869m	29 July 2009	VL & NS
*karindus*	M	W340		h01/GH5	KT582732	Kordestan	Dare Dozdan 35°52.05'N; 046°33.03'E	2066m	30 July 2009	VL & NS
*karindus*	M	W341		h01/GH5	KT582733	Kordestan	Dare Dozdan 35°52.05'N; 046°33.03'E	2066m	30 July 2009	VL & NS
*karindus*	M	W342		h01/GH5	KT582734	Kordestan	Dare Dozdan 35°52.05'N; 046°33.03'E	2066m	30 July 2009	VL & NS
*karindus*	M	W354		h04/GH5	KT582737	Kordestan	Dare Dozdan 35°52.05'N; 046°33.03'E	2277m	31 July 2009	VL & NS
*karindus*	M	W355		h01/GH5	KT582735	Kordestan	Dare Dozdan 35°52.05'N; 046°33.03'E	2277m	31 July 2009	VL & NS
*karindus*	M	W361		h01/GH5	KT582736	Kordestan	Dare Dozdan 35°52.05'N; 046°33.03'E	2066m	31 July 2009	VL & NS
*karindus*	M	W366		h01/GH5	KT582738	Kordestan	Dare Dozdan 35°52.05'N; 046°33.03'E	2066m	31 July 2009	VL & NS
*karindus*	M	V069		h01/GH5	KT582739	Kordestan	Dare Dozdan 35°51.30'N; 046°42.60'E	2200m	27 July 2014	NS & AB
*karindus*	M	V070		h01/GH5	KT582740	Kordestan	Dare Dozdan 35°51.30'N; 046°42.60'E	2200m	27 July 2014	NS & AB
*karindus*	M	W370	73	h05/GH4	KT582722	Lorestan	Nahavand 34°02.57'N; 048°20.22'E	2173m	2 August 2009	VL & NS
*karindus*	M	W371	73	h05/GH4	KT582723	Lorestan	Nahavand 34°02.57'N; 048°20.22'E	2173m	2 August 2009	VL & NS
*karindus*	M	W372	73	h09/GH2	KT582724	Lorestan	Nahavand 34°02.57'N; 048°20.22'E	2173m	2 August 2009	VL & NS
*karindus*	M	W373	73	h05/GH4	KT582725	Lorestan	Nahavand 34°02.57'N; 048°20.22'E	2173m	2 August 2009	VL & NS
*karindus*	M	W374		h09/GH2	KT582726	Lorestan	Nahavand 34°02.57'N; 048°20.22'E	2173m	2 August 2009	VL & NS
*karindus*	M	W375		h09/GH2	KT582727	Lorestan	Nahavand 34°02.57'N; 048°20.22'E	2173m	2 August 2009	VL & NS
*karindus*	M	W376		h09/GH2	KT582728	Lorestan	Nahavand 34°02.57'N; 048°20.22'E	2173m	2 August 2009	VL & NS
*karindus*	M	W388		h08/GH2	KT582731	Lorestan	Nahavand 34°02.57'N; 048°20.22'E	1950–2173m	3 August 2009	VL & NS
*karindus*	M	W389		h05/GH4	KT582729	Lorestan	Nahavand 34°02.57'N; 048°20.22'E	1950–2173m	3 August 2009	VL & NS
*karindus*	M	W390		h09/GH2	KT582730	Lorestan	Nahavand 34°02.57'N; 048°20.22'E	1950–2173m	3 August 2009	VL & NS
*karindus*	M	W391				Lorestan	Nahavand 34°02.57'N; 048°20.22'E	1950–2173m	3 August 2009	VL & NS
*karindus*	M	W392				Lorestan	Nahavand 34°02.57'N; 048°20.22'E	1950–2173m	3 August 2009	VL & NS
*karindus*	M	U217				Lorestan	Nahavand, 34°02.92'N; 48°20.40'E	2161 m	19 July 2011	VL & NS
*karindus*	M	U218				Lorestan	Nahavand, 34°02.92'N; 48°20.40'E	2161 m	19 July 2011	VL & NS
*karindus*	M	U219				Lorestan	Nahavand, 34°02.92'N; 48°20.40'E	2161 m	19 July 2011	VL & NS
*karindus*	M	U220				Lorestan	Nahavand, 34°02.92'N; 48°20.40'E	2161 m	19 July 2011	VL & NS
*karindus*	M	U223				Lorestan	Nahavand, 34°02.92'N; 48°20.40'E	2161 m	19 July 2011	VL & NS
*karindus*	M	U228				Lorestan	Nahavand, 34°02.91'N; 48°21.08'E	2020 m	20 July 2011	VL & NS
*karindus*	M	U229				Lorestan	Nahavand, 34°02.91'N; 48°21.08'E	2020 m	20 July 2011	VL & NS
*karindus*	M	U230				Lorestan	Nahavand, 34°02.91'N; 48°21.08'E	2020 m	20 July 2011	VL & NS
*karindus*	M	U231				Lorestan	Nahavand, 34°02.91'N; 48°21.08'E	2020 m	20 July 2011	VL & NS
*karindus*	M	U232				Lorestan	Nahavand, 34°02.91'N; 48°21.08'E	2020 m	20 July 2011	VL & NS
*karindus*	M	U233				Lorestan	Nahavand, 34°02.91'N; 48°21.08'E	2020 m	20 July 2011	VL & NS
*karindus*	M	U234				Lorestan	Nahavand, 34°02.91'N; 48°21.08'E	2020 m	20 July 2011	VL & NS
*karindus*	M	U235				Lorestan	Nahavand, 34°02.91'N; 48°21.08'E	2020 m	20 July 2011	VL & NS
*karindus*	M	U236				Lorestan	Nahavand, 34°02.91'N; 48°21.08'E	2020 m	20 July 2011	VL & NS
*karindus*	M	U237				Lorestan	Nahavand, 34°02.91'N; 48°21.08'E	2020 m	20 July 2011	VL & NS
*karindus*	M	U238				Lorestan	Nahavand, 34°02.91'N; 48°21.08'E	2020 m	20 July 2011	VL & NS
*karindus*	M	U239				Lorestan	Nahavand, 34°02.91'N; 48°21.08'E	2020 m	20 July 2011	VL & NS
*karindus*	M	U240				Lorestan	Nahavand, 34°02.91'N; 48°21.08'E	2020 m	20 July 2011	VL & NS
*karindus*	M	U256				Lorestan	Nahavand, 34°02.91'N; 48°21.08'E	2020 m	20 July 2011	VL & NS
*karindus*	M	U257				Lorestan	Nahavand, 34°02.91'N; 48°21.08'E	2020 m	20 July 2011	VL & NS
*karindus*	M	U262				Lorestan	Nahavand, 34°02.91'N; 48°21.08'E	2020 m	20 July 2011	VL & NS
*karindus*	M	U263				Lorestan	Nahavand, 34°02.91'N; 48°21.08'E	2020 m	20 July 2011	VL & NS
*karindus*	M	U264				Lorestan	Nahavand, 34°02.91'N; 48°21.08'E	2020 m	20 July 2011	VL & NS
*karindus*	M	U265				Lorestan	Nahavand, 34°02.91'N; 48°21.08'E	2020 m	20 July 2011	VL & NS
*karindus*	M	U266				Lorestan	Nahavand, 34°02.91'N; 48°21.08'E	2020 m	20 July 2011	VL & NS
*karindus*	M	U267				Lorestan	Nahavand, 34°02.91'N; 48°21.08'E	2020 m	20 July 2011	VL & NS
*karindus*	M	U278				Lorestan	Nahavand, 34°02.91'N; 48°21.08'E	2020 m	20 July 2011	VL & NS
*karindus*	M	U279				Lorestan	Nahavand, 34°02.91'N; 48°21.08'E	2020 m	20 July 2011	VL & NS
*karindus*	M	U280				Lorestan	Nahavand, 34°02.91'N; 48°21.08'E	2020 m	20 July 2011	VL & NS
*karindus*	M	U281				Lorestan	Nahavand, 34°02.91'N; 48°21.08'E	2020 m	20 July 2011	VL & NS
*karindus*	M	Z381	73	h09/GH2	KT582691	Lorestan	W of Borujerd, Kuh-e Garin mount.Vennnai, 33°53.89'N; 48°34,03'E	2150m	21 July 2007	VL & NS
*karindus*	M	Z382	73	h09/GH2	KT582692	Lorestan	W of Borujerd, Kuh-e Garin mount.Vennnai, 33°53.89'N; 48°34,03'E	2150m	21 July 2007	VL & NS
*karindus*	M	Z396	73	h09/GH2	KT582693	Lorestan	W of Borujerd, Kuh-e Garin mount.Vennnai, 33°53.89'N; 48°34,03'E	2150m	21 July 2007	VL & NS
*karindus*	M	Z397	73	h10/GH2	KT582694	Lorestan	W of Borujerd, Kuh-e Garin mount.Vennnai, 33°53.89'N; 48°34,03'E	2150m	21 July 2007	VL & NS
*karindus*	M	Z398	73	h09/GH2	KT582695	Lorestan	W of Borujerd, Kuh-e Garin mount.Vennnai, 33°53.89'N; 48°34,03'E	2150m	21 July 2007	VL & NS
*karindus*	M	Z399	73	h11/GH1	KT582696	Lorestan	W of Borujerd, Kuh-e Garin mount.Vennnai, 33°53.89'N; 48°34,03'E	2150m	21 July 2007	VL & NS
*karindus*	M	Z400	73	h10/GH2	KT582697	Lorestan	W of Borujerd, Kuh-e Garin mount.Vennnai, 33°53.89'N; 48°34,03'E	2150m	21 July 2007	VL & NS
*karindus*	M	Z408	73	h10/GH2	KT582698	Lorestan	W of Borujerd, Kuh-e Garin mount.Vennnai, 33°53.89'N; 48°34,03'E	2150m	21 July 2007	VL & NS
*karindus*	M	Z412	73	h11/GH1	KT582700	Lorestan	W of Borujerd, Kuh-e Garin mount.Vennnai, 33°53.89'N; 48°34,03'E	2150m	22 July 2007	VL & NS
*karindus*	M	Z413				Lorestan	W of Borujerd, Kuh-e Garin mount.Vennnai, 33°53.89'N; 48°34,03'E	2150m	22 July 2007	VL & NS
*karindus*	M	Z416		h09/GH2	KT582699	Lorestan	W of Borujerd, Kuh-e Garin mount.Vennnai, 33°53.89'N; 48°34,03'E	2150m	22 July 2007	VL & NS
*karindus*	M	V331				Lorestan	W of Borujerd, Kuh-e Garin mount.Vennnai, 33°53.89'N; 48°34,03'E	2150m	2 August 2014	NS & AB
*karindus*	M	V335				Lorestan	W of Borujerd, Kuh-e Garin mount.Vennnai, 33°53.89'N; 48°34,03'E	2150m	2 August 2014	NS & AB
*karindus*	M	V336				Lorestan	W of Borujerd, Kuh-e Garin mount.Vennnai, 33°53.89'N; 48°34,03'E	2150m	2 August 2014	NS & AB
*karindus*	M	W061	73,74,75	h12/GH1	KT582711	Lorestan	Saravand, 33°22.39'N; 49°10.25'E	2070m	21 July 2009	VL & NS
*karindus*	M	W062	ca73			Lorestan	Saravand, 33°22.39'N; 49°10.25'E	2070m	22 July 2009	VL & NS
*karindus*	M	W063	71	h12/GH1	KT582712	Lorestan	Saravand, 33°22.39'N; 49°10.25'E	2070m	22 July 2009	VL & NS
*karindus*	M	W064	73	h12/GH1	KT582713	Lorestan	Saravand, 33°22.39'N; 49°10.25'E	2070m	22 July 2009	VL & NS
*karindus*	M	W065	ca73	h12/GH1	KT582714	Lorestan	Saravand, 33°22.39'N; 49°10.25'E	2070m	22 July 2009	VL & NS
*karindus*	M	W072	ca73	h12/GH1	KT582715	Lorestan	Saravand, 33°22.39'N; 49°10.25'E	2070m	22 July 2009	VL & NS
*karindus*	M	W073				Lorestan	Saravand, 33°22.39'N; 49°10.25'E	2070m	22 July 2009	VL & NS
*karindus*	M	W074				Lorestan	Saravand, 33°22.39'N; 49°10.25'E	2070m	22 July 2009	VL & NS
*karindus*	M	W075				Lorestan	Saravand, 33°22.39'N; 49°10.25'E	2070m	22 July 2009	VL & NS
*karindus*	M	W081				Lorestan	Saravand, 33°22.39'N; 49°10.25'E	2070m	22 July 2009	VL & NS
*karindus*	M	W082				Lorestan	Saravand, 33°22.39'N; 49°10.25'E	2070m	22 July 2009	VL & NS
*karindus*	M	W083				Lorestan	Saravand, 33°22.39'N; 49°10.25'E	2070m	22 July 2009	VL & NS
*karindus*	M	W084				Lorestan	Saravand, 33°22.39'N; 49°10.25'E	2070m	22 July 2009	VL & NS
*karindus*	M	W085				Lorestan	Saravand, 33°22.39'N; 49°10.25'E	2070m	22 July 2009	VL & NS
*karindus*	M	W086				Lorestan	Saravand, 33°22.39'N; 49°10.25'E	2070m	22 July 2009	VL & NS
*karindus*	M	W087				Lorestan	Saravand, 33°22.39'N; 49°10.25'E	2070m	22 July 2009	VL & NS
*karindus*	M	W093				Lorestan	Saravand, 33°22.39'N; 49°10.25'E	2070m	23 July 2009	VL & NS
*karindus*	M	W094				Lorestan	Saravand, 33°22.39'N; 49°10.25'E	2070m	23 July 2009	VL & NS
*karindus*	M	W095				Lorestan	Saravand, 33°22.39'N; 49°10.25'E	2070m	23 July 2009	VL & NS
*karindus*	M	W096				Lorestan	Saravand, 33°22.39'N; 49°10.25'E	2070m	23 July 2009	VL & NS
*karindus*	M	W377		h12/GH1	KT582716	Lorestan	Saravand, 33°22.39'N; 49°10.25'E	2100–2250m	3 August 2009	VL & NS
*karindus*	M	W378		h12/GH1	KT582717	Lorestan	Saravand, 33°22.39'N; 49°10.25'E	2100–2250m	3 August 2009	VL & NS
*karindus*	M	W379		h06/GH3	KT582718	Lorestan	Saravand, 33°22.39'N; 49°10.25'E	2100–2250m	3 August 2009	VL & NS
*karindus*	M	W380		h12/GH1	KT582719	Lorestan	Saravand, 33°22.39'N; 49°10.25'E	2100–2250m	3 August 2009	VL & NS
*karindus*	M	W381		h12/GH1	KT582720	Lorestan	Saravand, 33°22.39'N; 49°10.25'E	2100–2250m	3 August 2009	VL & NS
*karindus*	M	W382		h07/GH3	KT582721	Lorestan	Saravand, 33°22.39'N; 49°10.25'E	2100–2250m	3 August 2009	VL & NS
*karindus*	M	W383				Lorestan	Saravand, 33°22.39'N; 49°10.25'E	2100–2250m	3 August 2009	VL & NS
*karindus*	M	W386				Lorestan	Saravand, 33°22.39'N; 49°10.25'E	2100–2250m	3 August 2009	VL & NS
*karindus*	M	W387				Lorestan	Saravand, 33°22.39'N; 49°10.25'E	2100–2250m	3 August 2009	VL & NS
*karindus*	M	U168		h12/GH1	KT582741	Lorestan	Darreh Takht, 33°21.19'N; 49°22.34'E	2000–2100 m	18 July 2011	VL & NS
*karindus*	M	U178		h12/GH1	KT582742	Lorestan	Darreh Takht, 33°21.19'N; 49°22.34'E	2000–2100 m	18 July 2011	VL & NS
*karindus*	M	U179		h12/GH1	KT582743	Lorestan	Darreh Takht, 33°21.19'N; 49°22.34'E	2000–2100 m	18 July 2011	VL & NS
*karindus*	F	U169		h06/GH3	KT582744	Lorestan	Darreh Takht, 33°21.19'N; 49°22.34'E	2000–2100 m	18 July 2011	VL & NS

In addition, we used the following sequences from GenBank: ***COI***: AY557145 Polyommatus (Agrodiaetus) karindus (h01/GH5); AY557007 Polyommatus (Agrodiaetus) dama; AY556887 Polyommatus (Agrodiaetus) birunii.

Fresh (not worn) adult males were used to investigate the karyotypes. After capturing a butterfly in the field, it was placed in a glassine envelope for 1–2 hours to keep it alive until processed. Butterflies were killed by pressing the thorax. Testes for karyotype analysis were removed from the abdomen and placed into a 0.5 mL vial with a freshly prepared fixative (ethanol and glacial acetic acid 3:1). Then each wing was carefully removed from the body using forceps and placed into glassine envelope. The wingless body was placed into a plastic, 2 mL vial with pure 100% ethanol (for DNA analysis). Each vial with ethanol has already been numbered. This ID number was also used to label a vial with the fixative and a glassine envelope, in which the wings are preserved. Thus, each specimen was individually fixed. All collected specimens are kept in the Zoological Institute of the Russian Academy of Science (St. Petersburg) (ZIN RAS). All the testes are kept in the Department of Karyosystematics (ZIN RAS).

### Chromosome preparation and karyotyping

Testes were stored in the fixative for 1–12 months at 4 °C. Then the gonads were stained in 2% acetic orcein for 30–60 days at 18–20 °C. Chromosome preparations were obtained as previously described (Talavera et al. 2013b). Different stages of male meiosis were examined by using a light microscope (Amplival, Carl Zeiss). An original two-phase method of chromosome analysis was used (Lukhtanov et al. 2006).

### DNA Extraction and Sequencing

A fragment of the mitochondrial cytochrome *c* oxidase subunit I gene (first 690 positions) served as a mitochondrial molecular marker. Thoracic muscles and first abdominal segments were used for DNA extraction. The segments were homogenized in CTAB buffer and digested with proteinase K (10 mg/mL) for three hours at 60 °C. DNA was purified through successive ethanol precipitations and stored in dd H_2_O at -20 °C.

For DNA amplification of *COI* we used primers K698 and Nancy (Caterino and Sperling 1999). PCR reactions (50 µl) contained 10 pmol each of forward and reverse primer, 1 mM dNTPs, 10x PCR Buffer (0.01 mM Tris-HCl, 0.05 M KCl, 0.1% Triton X–100: pH 9.0), 1 unit Taq DNA Polymerase (Fermentas), 5 mM MgCl_2_ and were conducted using the following profile: initial 4 min denaturation at 94 °C and 30 cycles of 30 sec denaturation at 94 °C, 1 min annealing at 55 °C, 1 min extension at 72 °C and 5 min final elongation at 72 °C. PCR products were analyzed on 1.5% agarose gel, and purified using GeneJET PCR purification kit (Fermentas). Sequencing of double-stranded product was carried out at the Research Resource Center for Molecular and Cell Technologies (St. Petersburg State University).

### Sequence alignments and phylogeny inference

The sequences were edited and aligned using CHROMAS 2.4.3 (http://www.technelysium.com.au/), Geneious 8.1.6 (Kearse et al. 2012), and BioEdit 7.0.3 (Hall 2011) software. The alignment was unambiguous, as all the sequences were of equal length and included no insertions/deletions. Primer sequences were cropped. This resulted in final alignment of 690 bp *COI* fragments. The analysis involved *COI* sequences inferred from 54 Polyommatus (Agrodiaetus) karindus specimens. Additional sequences of the Polyommatus (Agrodiaetus) dama (accession number AY557007) and Polyommatus (Agrodiaetus) karindus (accession number AY557145) were found in GenBank (Wiemers 2003) and were included into analysis, since these sequences completely overlapped with our fragment. We used sequence of Polyommatus (Agrodiaetus) birunii (Eckweiler & ten Hagen, 1998) (accession number AY556558) as an outgroup to root the phylogeny (according to available data, this species does not belong to the group closely related to Polyommatus (Agrodiaetus) dama). Thus, the final analysis included in total 57 *COI* sequences. A Bayesian approach for estimating phylogeny was used. Bayesian analyses were performed using the program MrBayes 3.2 (Ronquist et al. 2012), with the nucleotide substitution model GTR+G+I as suggested by jModelTest (Posada 2008). TRACER, v. 1.4 was used for summarizing the results of Bayesian phylogenetic analyses (http://beast.bio.ed.ac.uk/Tracer). A maximum–parsimony haplotype network was built using TCS v. 1.21, with a 99% parsimony connection limit (Clement et al. 2000).

## Results

### Analysis of karyotypes

Meiotic karyotypes were studied in 48 specimens of Polyommatus (Agrodiaetus) karindus from different Iranian localities. Depending on karyotypes and localities, 2 groups of individuals can be distinguished (Table 1 and see below).

**Group I** (Polyommatus (Agrodiaetus) karindus from NW Iran)

**Figure 2.**
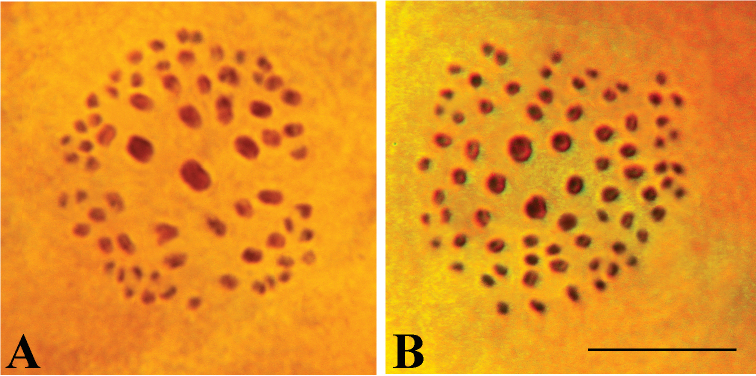
Male meiosis I karyotypes of: **A** Polyommatus (Agrodiaetus) karindus karindus, sample E399, Iran, Kordestan, 40 km SW Saqqez, 1800–1900 m, 2004.VII.29, V. Lukhtanov leg., n = 68 **B** Polyommatus (Agrodiaetus) karindus saravandi, sample W372, Iran, Nahavand 34°02.57'N; 048°20.22'E, 2173m, 2009.VIII.02, V. Lukhtanov & N. Shapoval leg., n = 73. Scale bar = 10 µm.

The haploid chromosome number n = 68 was found in meiotic metaphase I (MI) and meiotic metaphase II (MII) cells. The MI karyotype displayed 5 large bivalents in the center of metaphase plate and 63 smaller bivalents in the periphery (Fig. 2A).

**Group II** (will be described below as Polyommatus (Agrodiaetus) karindus saravandi from central Iran)

The haploid chromosome number n = 73 was found in meiotic MI and MII cells of studied individuals (Fig. 2B). The MI karyotype was strongly asymmetric with 5–6 larger bivalents in the center of the MI plate and 67–68 smaller bivalents in the periphery.

### Phylogenetic analysis of molecular data

**Figure 3.**
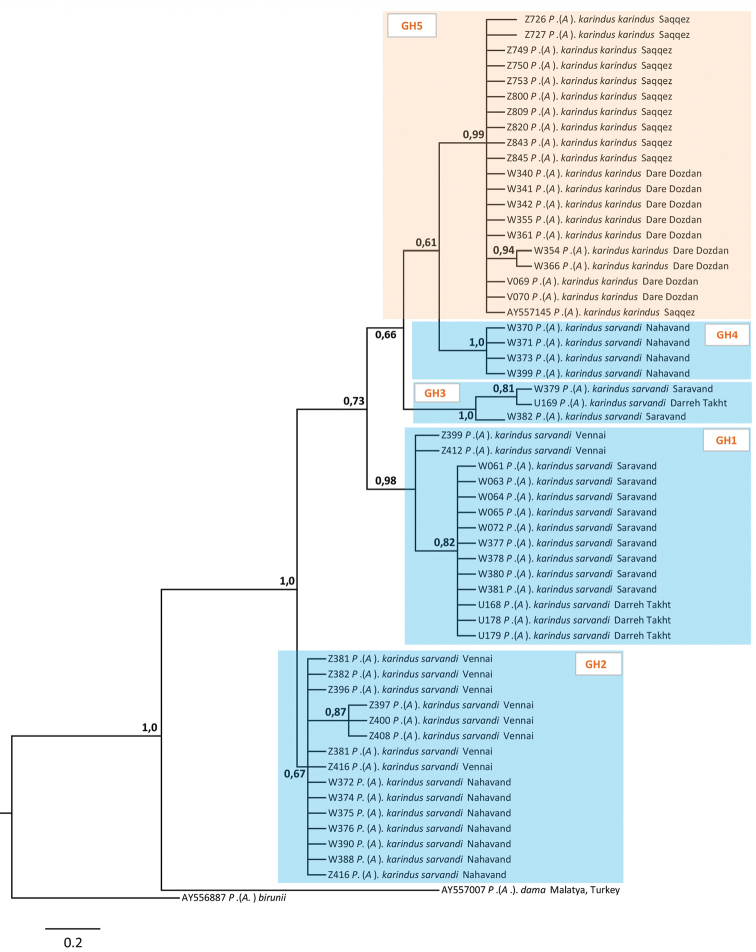
The Bayesian tree of Polyommatus (Agrodiaetus) dama and Polyommatus (Agrodiaetus) karindus based on analysis of the cytochrome c oxidase subunit I gene from 57 specimens. Numbers at nodes indicate Bayesian posterior probability. *Agrodiaetus karindus karindus* and *Agrodiaetus karindus saravandi* clusters highlighted in pink and blue respectively.

**Figure 4.**
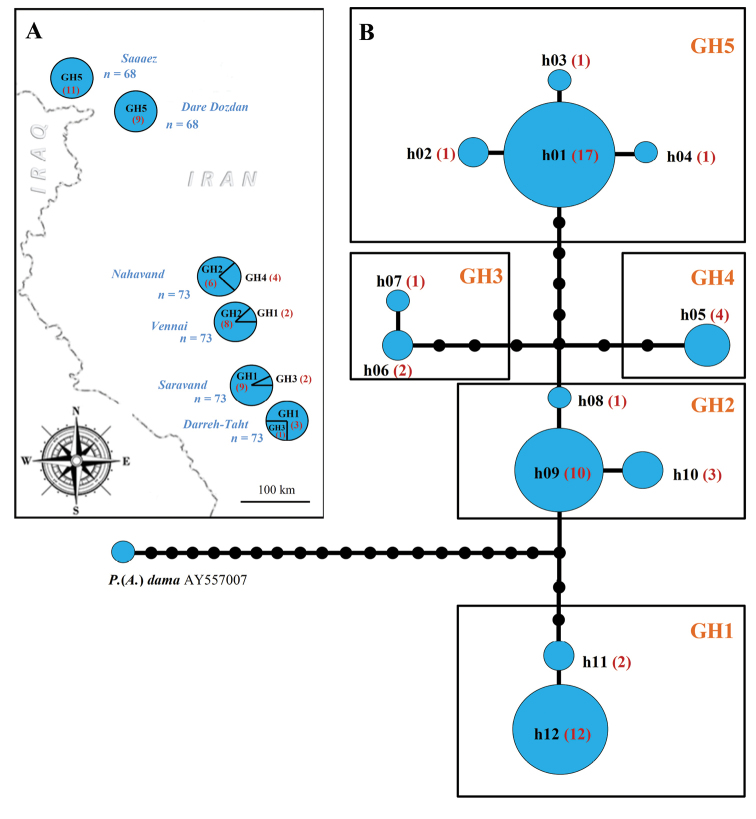
*COI* Haplotype analysis. **A** geographical distribution of haplogroups. Number of studied individuals sharing the same haplogroup is given in parentheses **B** most parsimonious *COI* haplotype network; h01–h12 are *COI* haplotypes; GH1–GH5 are *COI* haplogroups. Number of studied individuals sharing the same haplotype is given in parentheses.

A Bayesian inference recovered Polyommatus (Agrodiaetus) karindus as a strongly supported monophyletic clade characterized by a specific set of fixed nucleotide substitutions (Fig. 3). Specimens of Polyommatus (Agrodiaetus) karindus were divided into several clusters: one cluster united specimens of Polyommatus (Agrodiaetus) karindus collected in north–west Iran (Fig. 3, GH5, highlighted in pink) and the others (Fig. 3, GH1–GH4, highlighted in blue) included specimens of central Iran populations (described here as a novel subspecies Polyommatus (Agrodiaetus) karindus saravandi). Most parsimonious *COI* haplotype network demonstrated similar pattern (Fig. 4). Polyommatus (Agrodiaetus) dama differs from Polyommatus (Agrodiaetus) karindus by at least 20 fixed nucleotide substitutions. Specimens of Polyommatus (Agrodiaetus) karindus form several haplotypes clustered in five different haplogroups. In general, composition of each haplogroup reflects geographical distribution of butterflies. Thus, majority of the specimens from easternmost (Saravand and Darreh Takht) and central west (Vennai, Nahavand) localities form two distinct haplogroups: GH1 and GH2. Nevertheless, two specimens from Vennai (approx. 80 km NW from Saravand) were found to have mitochondrial haplotype similar to that in easternmost populations, which has led to the suggestion that there is no complete isolation (reproductive or/and geographical) between population from Vennai and easternmost populations. The third haplogroup (GH3) consists of only three specimens, which were collected in Saravand and Darreh Takht. Interestingly, the third haplogroup differs drastically (by 10–12 fixed nucleotide substitutions) from the haplotypes, which comprise all other specimens from Saravand and Darreh Takht (group GH1). The fourth haplogroup (GH4) unites four specimens from Nahavand. Finally, all the haplotypes found in NW Iran constituted a subset of the distinct haplogroup (GH5). Thus, most parsimonious *COI* haplotype network reflects complex phylogeographic pattern of Polyommatus (Agrodiaetus) karindus.

## Discussion

We have found that a taxon usually identified as Polyommatus (Agrodiaetus) karindus is represented in Iran by two geographically separated groups of individuals. The first group unites specimens collected in NW Iran, while the second group comprises specimens from central Iran. The representatives of these groups have different chromosome numbers, n = 68 and n = 73 respectively. They also have at least eight fixed nucleotide differences in 690 bp fragment of mitochondrial *COI* gene. The first group is monophyletic with respect to both *COI* gene and karyotype (n = 68). The second group has complicated genetic structure, comprises several differentiated populations and is paraphyletic with respect to the *COI* gene. Despite this gene paraphyly, it appears as a clearly monophyletic group with respect to its karyotype (n = 73). Thus, the NW and central Iranian groups are differentiated by at least five fixed chromosome fusions/fissions. Fixed chromosome differences are often considered as characters associated with reproductive isolation (King 1993). From this point of view, the NW and central Iranian groups could be theoretically treated as a different species. However, our recent studies on *Agrodiaetus* demonstrated that multiple chromosome fusions and fissions did not block fertility in chromosomal hybrids (Lukhtanov et al. 2015b). In other words, differentiation by five fixed chromosome rearrangements would not guarantee impossibility of blending populations together when they occur in sympatry. Thus, NW and central Iranian groups of populations should be considered as a subspecies rather than separate species.

Since Polyommatus (Agrodiaetus) karindus (Riley, 1921) (orig. comb. Lycaena dama subsp. karinda) was described from NW Iran (type locality is “Harir, Karind, and Karind Gorge, N.W. Persia” according to original description, and “N.W. Persia, Karind Gorge, 6000 ft” according to lectotype designation made by Bálint (1999) (not from central Iran), the name *Polyommatus karindus karindus* should be attributed to the NW Iranian group of populations. The formal description and naming of the central Iranian group is provided below.

### Description of the novel taxon

#### Polyommatus (Agrodiaetus) karindus saravandi ssp. n.

Taxon classificationAnimaliaLepidopteraLycaenidae

http://zoobank.org/ADC4F3C8-B804-4869-955A-9F7C7164B0C5

**Figure 5.**
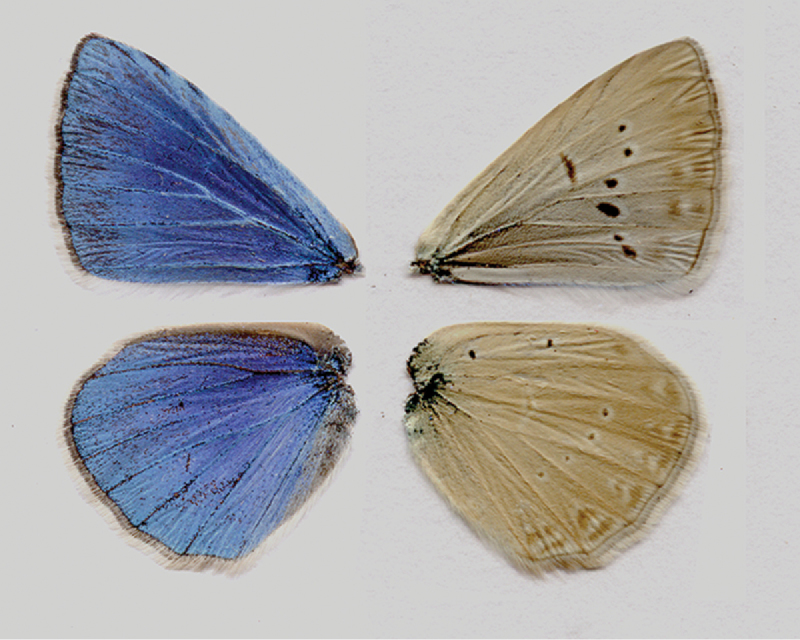
Holotype of Polyommatus (Agrodiaetus) karindus saravandi, sample W064. Upperside (left) and underside (right) of the male wings.

**Figure 6.**
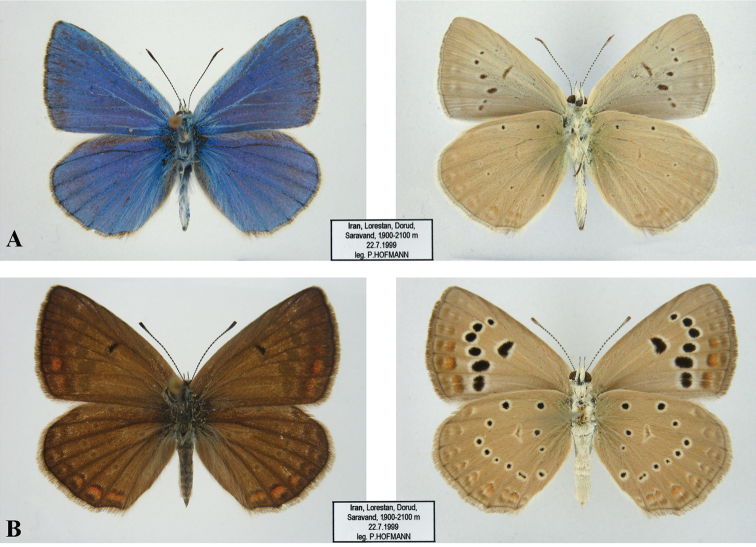
Underside and upperside of the Polyommatus (Agrodiaetus) karindus saravandi ssp. n. wings. **A** upperside (left) and underside (right) of the male wings **B** upperside (left) and underside (right) of the female wings.

Fig. 1 – map, Fig. 2B karyotype, Figs 3 , 4 phylogeny, Fig. 5 , Fig. 6A, B – Underside and upperside of the male and female wings

##### Holotype.

♂. Forewing length 34.0 mm. Iran, Lorestan province, Zagros Mt., vicinity of Saravand village, 33°22.39'N; 49°10.25'E, 2070 m, 22.07 2009. N. Shapoval and V. Lukhtanov leg. In the Zoological Institute of the Russian Academy of Sciences (St. Petersburg). Specimen field code W064, GenBank code for mitochondrial cytochrome *c* oxidase subunit I (*COI*) gene (partial cds) is KT582713.

##### Paratypes.

87 ♂♂, field codes W061, W062, W063, 21.07.2009; W065, W072, W073, W074, W075, W081, W082, W083, W084, W085, W086, W087, the same locality, date and collectors as the holotype. Field codes W093, W094, W095, W096 23.07.2009, the same locality and collectors as the holotype. Field codes W377, W378, W379, W380, W381, W382, W383, W386, W387 03.08.2009, the same locality and collectors as the holotype. Field codes W370, W371, W372, W373, W374, W375, W376, Iran, Lorestan province, Zagros Mt., vicinity of Nahavand village, 34°02.57'N; 048°20.22'E, 2170 m, 02.08.2009, the same collectors as the holotype. Field codes W388, W389, W390, W391, W392, Iran, Lorestan province, Zagros Mt., vicinity of Nahavand village, 34°02.57'N; 048°20.22'E, 2170 m, 02.08.2009, the same collectors as the holotype. Field codes U217, U218, U219, U220, U223, Iran, Lorestan province, Zagros Mt., vicinity of Nahavand village, 34°02.57'N; 048°20.22'E, 2170 m, 19.07.2011, the same collectors as the holotype. Field codes U228, U229, U230, U231, U232, U233, U234, U235, U236, U237, U238, U239, U240, U256, U257, U262, U263, U264, U265, U266, U267, U278, U279, U280, U281, Iran, Lorestan province, Zagros Mt., vicinity of Nahavand village, 34° 02.92'N; 48° 20.40'E, 2160 m, 20.07.2011 the same collectors as the holotype. Field codes Z381, Z382, Z396, Z397, Z398, Z399, Z400, Z408, Iran, Lorestan province, Zagros Mt., W of Borujerd, Kuh-e Garin mount mount., Vennnai, 33°53.89'N; 48°34.03'E, 2150 m, 21.07.2007, the same collectors as the holotype. Field codes Z412, Z413, Z416, Iran, Lorestan province, Zagros Mt., W of Borujerd, Kuh-e Garin mount., Vennnai, 33°53.89'N; 48°34.03'E, 2150 m, 22.07.2007, the same collectors as the holotype. Field codes V331, 335, V336, Iran, Lorestan province, Zagros Mt., W of Borujerd, Kuh-e Garin mount., Vennnai, 33°53.89'N; 48°34.03'E, 2150 m, 02.08.2014, N. Shapoval and A. Barabanov leg. Field codes U169, U178, U179, Iran, Lorestan province, Zagros Mt., Darreh Takht, 33° 21.19'N; 49° 22.34'E, 2000–2100 m, 18.07.2011, the same collectors as the holotype. 1 ♀, field code U169 Iran, Lorestan province, Zagros Mt., Darreh Takht, 33° 21.19'N; 49° 22.34'E, 2000–2100 m, 18.07.2011, same collectors as the holotype. All paratypes are kept in the Zoological Institute of the Russian Academy of Sciences (St. Petersburg). GenBank accession numbers of the paratypes are presented in the Table 1.

##### *Derivatio nominis*.

The new taxon is named after the village Saravand, one of the places where it was found.

##### Description.

*Male upperside.* Forewing length 30–36 mm, ground colour bright blue with azure tint. Discoidal, submarginal and antemarginal marking absent on both fore- and hindwings. Black outer marginal line on forewings and hindwings very narrow; forewing hind margin with long white pubescence. Fringes of both wings dark grey; tips of hindwings veins indicated with fine black.

*Male underside.* Ground colour light grey, white streak on the hindwings absent. Basal black spots present only on hindwings. Discoidal series of spots present on fore- and hindwings, although the black spots composing it are minute. Postdiscal black marking very narrow, longitudinal, present only on forewings. Submarginal and marginal lunules only faintly indicated.

*Female upperside.* Ground colour brown with vastly darker veins. Discoidal black spots present on forewings. Submarginal markings dark brown with orange submarginal lunules well developed on forewing and hindwing. Fringe greyish-brown.

*Female underside.* General design as in males, but ground colour slightly darker.

*Genitalia.* The male genitalia have a structure typical for other species of the subgenus *Agrodiaetus* (Coutsis 1986). No specific characters in genitalia are found.

##### Diagnosis.

Genetically Polyommatus (Agrodiaetus) karindus saravandi differs from all other taxa of *Agrodiaetus* by fixed substitutions in mitochondrial gene *COI*. Phenotypically the new taxon is extremely similar to Polyommatus (Agrodiaetus) karindus karindus from north-west Iran, but they have different chromosome numbers, n=73 and n = 68 respectively.

##### Distribution.

Central part of Zagros Mountains, Iran.

##### Flight period.

From July to August.

##### Ecology.

Dry slopes, gorges and plateaus with xerophyte or steppe vegetation, sometimes wooded areas from 1800 up to 2800 m. Butterflies fly together with Polyommatus (Agrodiaetus) alcestis (Zerny, 1932), Polyommatus (Agrodiaetus) cyaneus (Staudinger, 1899), Polyommatus (Agrodiaetus) hamadanensis (de Lesse, 1959), Polyommatus (Agrodiaetus) lorestanus (Eckweiler, 1997) and Polyommatus (Agrodiaetus) zarathustra (Eckweiler, 1997).

## Supplementary Material

XML Treatment for Polyommatus (Agrodiaetus) karindus saravandi
